# Supplementary material for: p-Aminophenylalanine Involved in the Biosynthesis of Antitumor Dnacin B1 for Quinone Moiety Formation
Source: Molecules. 2020 Sep 12;25(18):4186. doi: 10.3390/molecules25184186 (PMC7570522; doi:10.3390/molecules25184186)
Supplement: Supplementary file 1 [file molecules-25-04186-s001.pdf]

molecules

p-Amino-phenylalanine Involved in the Biosynthesis of Antitumor Dnacin B1 for Quinone Moiety Formation

Xiaojing Hu[1], Xing Li[1], Yong Sheng[1], Hengyu Wang[1], Xiaobin Li[1,2], Dongjin Leng[1], Yixin Ou[1], Zixin Deng[1], Linquan Bai[1] and Qianjin Kang[1]*

[1]State Key Laboratory of Microbial Metabolism, Joint International Research Laboratory of Metabolic & Developmental Sciences, and School of Life Sciences & Biotechnology, Shanghai Jiao Tong University, Shanghai 200240, China.

[2]Zhuhai Precision Medical Center, Zhuhai People's Hospital, Zhuhai Hospital Affiliated with Jinan University, Zhuhai, Guangdong 519000, China.

*** Correspondence:**

*Dr. Qianjin Kang: qjkang@sjtu.edu.cn; Tel/Fax: +86 21 34206119

Supplementary Tables

Table S1. Strains and plasmids used in this study

Strains and plasmids	Description	Source or reference
Actinosynnema pretiosum		
A. pretiosum DSM 44131^T	Wild-type producer for dnacin B1	This work
HXJA01	Mutant of *din*-disruption	This work
Escherichia coli		
E. coli DH10B	Plasmid amplification strain	Invitrogen
E. coli ET12567/ pUZ8002	Conjugation donor strain	(Paget et al. 1999)
E. coli BL21(DE3)	Protein expression strain	Invitrogen
HXJE03	BL21(DE3) with plasmids pJQK350 and pJQK351	This work
HXJE04	BL21(DE3) with plasmids pCDFDuet and pETDuet	This work
Plasmids		
pBluescript SK (+)	*ColE, lacZ, bla, orif1*	Stratagene
pJTU1278	*rep pIJ101, lacZ, tsr, bla, oriT*	(He et al. 2010)
pJQK301	Construct for *din*-disruption	This work
pJQK302	pJQK301 with cloned *aac(3)IV*	This work

pET28a	*rep pBR322*, P_{T7}, His_6-tag, *kan*	Novagen
pCDFDuet	*rep CDF*, P_{T7}, *aadA*	Novagen
pETDuet	*rep pBR322*, P_{T7}, *bla*	Novagen
pJQK354	pCDFDuet with *dinV* gene	This work
pJQK355	pETDuet with *dinE* and *dinF* gene	This work
pJQK356	pET28a with *apat1* gene	This work
pJQK357	pET28a with *apat2* gene	This work
pJQK358	pET28a with *apat3* gene	This work
pJQK359	pET28a with *apat4* gene	This work
pJQK360	pET28a with *apat5* gene	This work
pJQK361	pET28a with *apat6* gene	This work
pJQK362	pET28a with *apat7* gene	This work
pJQK363	pET28a with *apat8* gene	This work
pJQK364	pET28a with *apat9* gene	This work
pJQK365	pET28a with *tyrB* gene	This work

Table S2. Primers used in this study

Primers	Sequences (5′→3′)
Primers used for the construction of knockout mutants	
din-del-L/F (*Eco*RI)	AGAATTCAGCGGTTGTGGGTGCTGTGC
din-del-L/R (*Hin*dIII)	AAAGCTTGGTGGTCTGGCGGTGTTCGA
din-del-R/F (*Hin*dIII)	AAAGCTTGTGGTTCCTGTGCCAGTTGGAG
din-del-R/R (*Kpn*I)	AGGTACCGGTGTCGATCCCCGTTCC
din-val/F	GGTGCTGCGCGAGGTGGA
din-val/R	CCCGGACACGTCGACCAC
din-in/F	ACGACACGTCTGATTCGGAG
din-in/R	CACAGCTCACCCAGGAACAC
Primers used for qPCR	
dinI-RT/F	CTCATCCGCCCCGACA
dinI-RT/R	GCCCGAACAGGACCGC
dinP-RT/F	TCAAGCAGTCAGCCACAGG
dinP-RT/R	CAGCGACAGCGAACCG
dinQ-RT/F	CGCTTCACCGGTTTCGC
dinQ-RT/R	GAGCACCTTCGGGTTGTCG
dinX-RT/F	CGACCACGAGAAGATGCG

dinX-RT/R	GAACCGGGACAGCCACA
dinY-RT/F	GTCTACCTGGTGCAGGGCA
dinY-RT/R	GGTGAAGCGGGTCTCGTC
hrdB-RT/F	GCGGTCGAGAAGTTCGACTA
hrdB-RT/R	CCGGGATGCGGATGGTG
Primers used for gene cloning and protein expression of functional genes	
dinV/F (*Bam*HI)	AGGATCCATGCGGACGCTGCTCATCGA
dinV/R (*Hin*dIII)	AAAGCTTTCATGGGATCCTCCGGGGT
dinE/F (*Bam*HI)	AGGATCCTTGTCGGTGGCTG
dinE/R (*Hin*dIII)	AAAGCTTAATTACTCTCTGG
dinF/F (*Nde*I)	AACATATGGTGCCCCTTGAGC
dinF/R (*Xho*I)	AACTCGAGGCTCTTCGGGGTC
APAT1/F (*Nde*I)	ACATATGGTGCGGGTACCAGCGCT
APAT1/R (*Eco*RI)	GAATTCCTACCCGCCCAGCTTCCTG
APAT2/F (*Nde*I)	ACATATGATGCAGCACTCGATCC
APAT2/R (*Eco*RI)	GAATTCTCAAGCCACCGGCACCTC
APAT3/F (*Nde*I)	ACATATGATGCTCGGTGTCTCCAC
APAT3/R (*Eco*RI)	GAATTCTCAACGCGCGGGCGGGGC
APAT4/F (*Nde*I)	ACATATGATGACCGCACCCGGACAG

APAT4/R (*Eco*RI)	GAATTCTCATGCGGTGTCGGGCGAGG
APAT5/F (*Nde*I)	ACATATGATGGCTGAGCCTGAGAC
APAT5/R (*Eco*RI)	GAATTCCTACTTCACCTCGGCGAGC
APAT6/F (*Nde*I)	ACATATGGTGGCCCGGATCGTCCAT
APAT6/R (*Eco*RI)	GAATTCCTACCCGTTGGCGGTGAAGG
APAT7/F (*Nde*I)	ACATATGATGAGCCCCGCGCTGGAC
APAT7/R (*Eco*RI)	GAATTCTCACCGGCTGACCTCCTTGCT
APAT8/F (*Nde*I)	ACATATGATGAGCGTGCGCACCCG
APAT8R (*Eco*RI)	GAATTCTCAGCGCTCGAACGACCG
APAT9/F (*Nde*I)	ACATATGGTGCGCTCCCTTTCCGTG
APAT9/R (*Eco*RI)	GAATTCTCACAGCACCTCCCCCAA
tyrB/F (*Nde*I)	AACATATGGTGTTTCAAAAAGTTG
tyrB/R (*Eco*RI)	AAGAATTCCATCACCGCAGC

Table S3. General genomic features of *Actinosynnema pretiosum* subsp. *auranticum* DSM 44131^{T}

Features	Chromosome
Genome topology	Circular
Assembly size (bp)	8,105,537
G + C content (%)	73.95
Protein coding genes	6663
tRNA genes	57
rRNA genes	7
Secondary metabolite gene clusters	28
NCBI BioProject	PRJNA541432
GenBank accession	JABBHD000000000.1

Table S4. AntiSMASH analysis of secondary metabolite BGCs of strains DSM 44131^T, X47, DSM 43827^T and ATCC 31280 genome sequences.

Type	DSM 44131^T	X47	DSM 43827^T	ATCC 31280
Terpene	4	4	4	4
Bacteriocin	1	1	1	1
Lanthipeptide	1	2	1	2
Indole	1	1	1	1
Type I PKS	8	8	8	8
NRPS	7	5	4	5
PKS-NRPS	2	2	3	1
Nucleoside	1	1	1	1
Lassopeptide	1	1	2	1
Thiopeptide	1	1	1	1
Oligosaccharide	1	1	0	0
Total	28	27	26	25

Table S5. Similarity analysis of the predictable BGCs among strains DSM 44131^T, X47, DSM 43827^T and ATCC 31280 genomes with known clusters.

Cluster	Type	Most similar known cluster (compound / strain)	DSM 44131^T	X47	DSM 43827^T	ATCC 31280
**1**	Terpene	Geosmin *Streptomyces coelicolor A3(2)*	+ 100%	+ 100%	+ 100%	+ 100%
**2**	Indole	Frankiamicin *Frankia* sp. *EAN1pec*	+ 14%	+ 14%	+ 14%	+ 14%
**3**	T1PKS	Amycolamycin *Amycolatopsis* sp. strain Hca4	+ 2%	+ 2%	+ 2%	+ 2%
**4**	T1PKS	Salinilactam *Salinispora tropica* CNB-440	+ 88%	+ 88%	+ 84%	+ 84%
**5**	Nucleoside	Tunicamycin *Streptomyces chartreusis* strain NRRL3882	+ 64%	+ 64%	+ 64%	+ 64%
**6**	NRPS-PKS	Nocamycin *Saccharothrix syringae* NRRL B-16468	+ 66%	+ 66%	-	-
**7**	PKS	Paulomycin *Streptomyces* sp. YN86	+ 32%	+ 32%	-	-

**8**	T1PKS	Ansamitocin<br>*Actinosynnema pretiosum* ATCC 31280	+<br>97%	+<br>97%	+<br>92%	+<br>95%
**9**	Lassop eptide	Cattlecin<br>*Streptomyces cattleya* NRRL 8057	+<br>50%	+<br>50%	+<br>50%	-
**10**	NRPS	Himastatin<br>*Streptomyces himastatinicus* ATCC 53653	+<br>8%	+<br>8%	-	-
**11**	NRPS	Naphthyridinomycin<br>*Streptomyces lusitanus*	+<br>64%	+<br>64%	-	-
**12**	Terpen e	Isorenieratene<br>*Streptomyces griseus* NBRC 13350	+<br>85%	+<br>85%	+<br>85%	+<br>85%
**13**	T1PKS	Coelimycin<br>*Streptomyces coelicolor* A3(2)	+<br>8%	+<br>8%	-	-
**14**	T1PKS	Kedarcidin<br>*Streptoalloteichus* sp. ATCC 53650	+<br>6%	+<br>6%	+<br>6%	+<br>6%
**15**	T1PKS	9-methylstreptimidone<br>*Streptomyces himastatinicus* ATCC 53653	+<br>6%	+<br>6%	-	-

**16**	Terpene	Phenalinolactone<br>*Streptomyces* sp. Tu6071	+<br>8%	+<br>8%	-	-
**17**	NRPS	Marformycins<br>*Streptomyces drozdowiczii* strain SCSIO 10141	+<br>20%	+<br>20%	-	-
**18**	T1PKS	Rifamycin<br>*Amycolatopsis mediterranei*	+<br>7%	+<br>7%	-	-
**19**	NRPS-PKS	Microtermolide<br>*Streptomyces* sp. MspMP-M5 B073DRAFT	+<br>33%	+<br>33%	+<br>26%	-
**20**	NRPS	Salinichelins<br>*Salinispora pacifica* CNY331	+<br>53%	+<br>53%	+<br>53%	+<br>53%
**21**	Terpene	SF2575<br>*Streptomyces* sp. SF2575	+<br>6%	+<br>6%	+<br>6%	+<br>6%
**22**	Lanthipeptide	Tetronasin<br>*Streptomyces longisporoflavus* strain NCIMB 11426	+<br>9%	+<br>9%	+<br>9%	+<br>9%
**23**	T1PKS	Macbecin<br>*Actinosynnema pretiosum* ATCC 31280	-	-	+<br>100%	+<br>100%
**24**	NRPS	Arylomycin	-	-	+	-

		Streptomyces filamentosus NRRL 11379			22%	
**25**	NRPS	Tallysomycin<br><br>*Streptoalloteichus hindustanus* strain ATCC 31158	-	-	+<br><br>50%	-
**26**	NRPS	Rimosamide<br><br>*Streptomyces rimosus* subsp. *rimosus* ATCC 10970	-	-	+<br><br>21%	+<br><br>21%
**27**	Terpen e	Brasilicardin A<br><br>*Nocardia brasiliensis*	-	-	+<br><br>45%	+<br><br>45%
**28**	T1PKS	Cyclizidine<br><br>*Streptomyces* sp. NCIB 11649	-	-	+<br><br>76%	-
**29**	NRPS	Nocardicin A<br><br>*Nocardia uniformis* subsp. *tsuyamanensis*	-	-	+<br><br>100%	+<br><br>100%
**30**	NRPS-PKS	Svaricin<br><br>*Streptomyces variabilis*	-	-	+<br><br>12%	+<br><br>12%

Table S6. The sequence analysis of nine APAT genes in strain DSM 44131^T

Gene	ORF (bp)	Location				Similarity with TyrB
		DSM 44131^T	X47	DSM 43827^T	ATCC 31280	Protein%
apat1	1173	Contig 6 (130,523-131,695)	7,778,946-7,780,118	7,909,287-7,910,459	7,999,330-8,000,502	30.5
apat2	1464	Contig 6 (147,276-145,813)	7,795,699-7,794,236	7,927,324-7,925,861	8,017,395-8,015,932	29.5
apat3	1167	Contig 6 (267,147-268,313)	7,916,736-7,915,570	8,031,576-8,030,410	8,122,829-8,121,663	34.4
apat4	1317	Contig 6 (474,018-475,334)	8,122,441-8,123,757	8,238,936-8,240,252	8,331,054-8,332,370	30.1
apat5	1242	Contig 10 (353,427-354,668)	7,626,785-7,628,026	7,777,575-7,778,816	7,867,491-7,868,732	31.1
apat6	1002	Contig 1 (885,098-886,099)	5,191,382-5,190,381	5,185,381-5,184,380	5,287,898-5,286,897	34.7
apat7	1146	Contig 3 (214,993-216,138)	6,602,979-6,601,834	6,746,083-6,744,932	6,836,045-6,834,894	35.1
apat8	1068	Contig 6 (638,667-639,734)	156,585-155,518	159,114-158,047	160,290-159,223	33.8
apat9	1134	Contig 3 (53,174-52,041)	6,440,015-6,438,882	6,524,023-6,522,890	6,680,124-6,678,991	34.8

Supplementary Figures

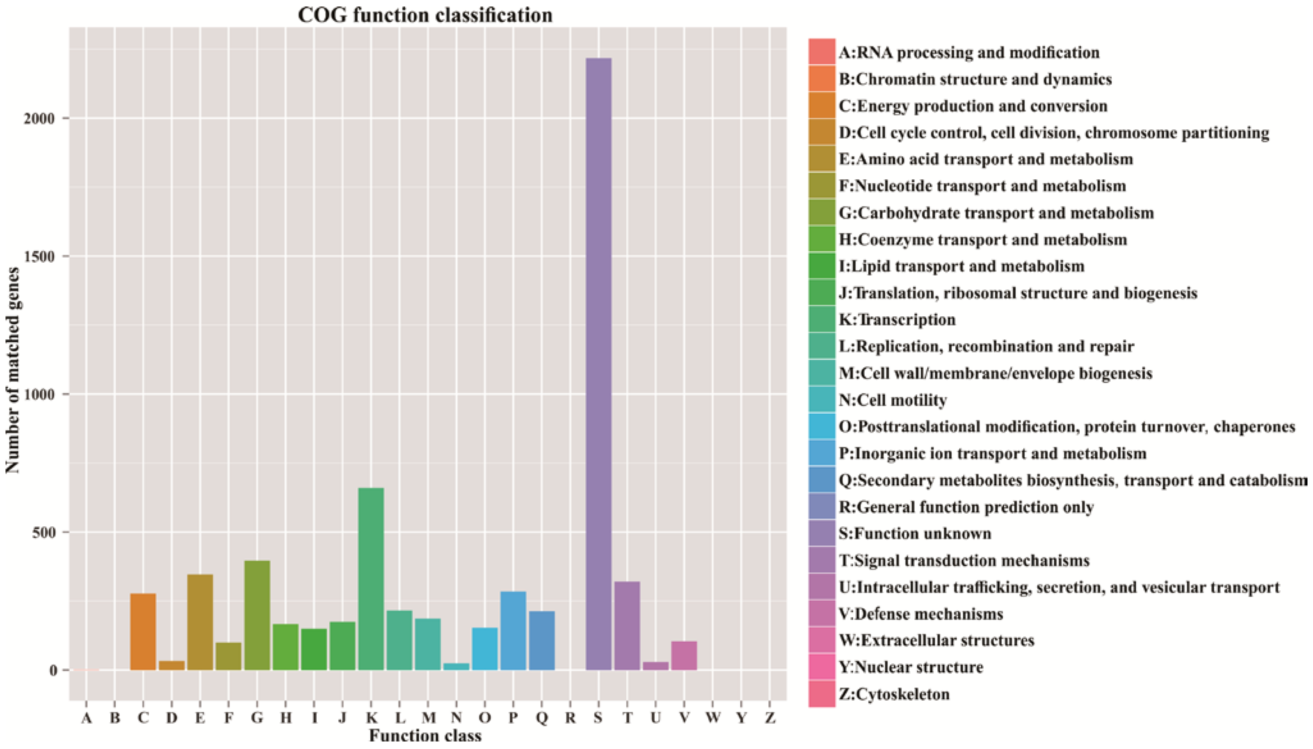

Figure S1. COG function classification of the annotated genes in the genome of strain DSM 44131^{T}. 6054 annotated ORFs are distributed into 25 COG functional categories. The horizontal axis and vertical axis represent COG functional categories and the matched gene numbers, respectively.

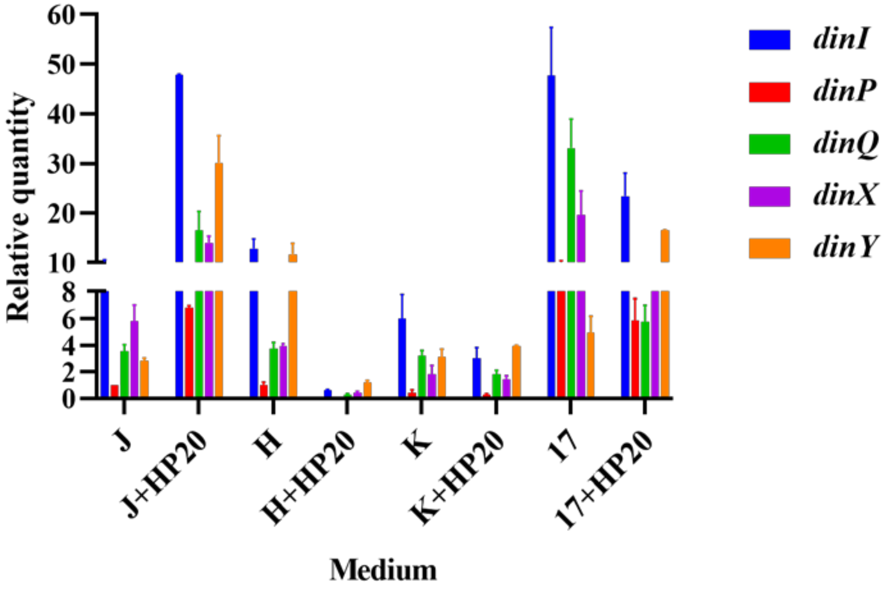

Figure S2. Transcriptional analysis of functional genes within dnacin B1 BGC in strain DSM 44131^{T} with different fermentation media.

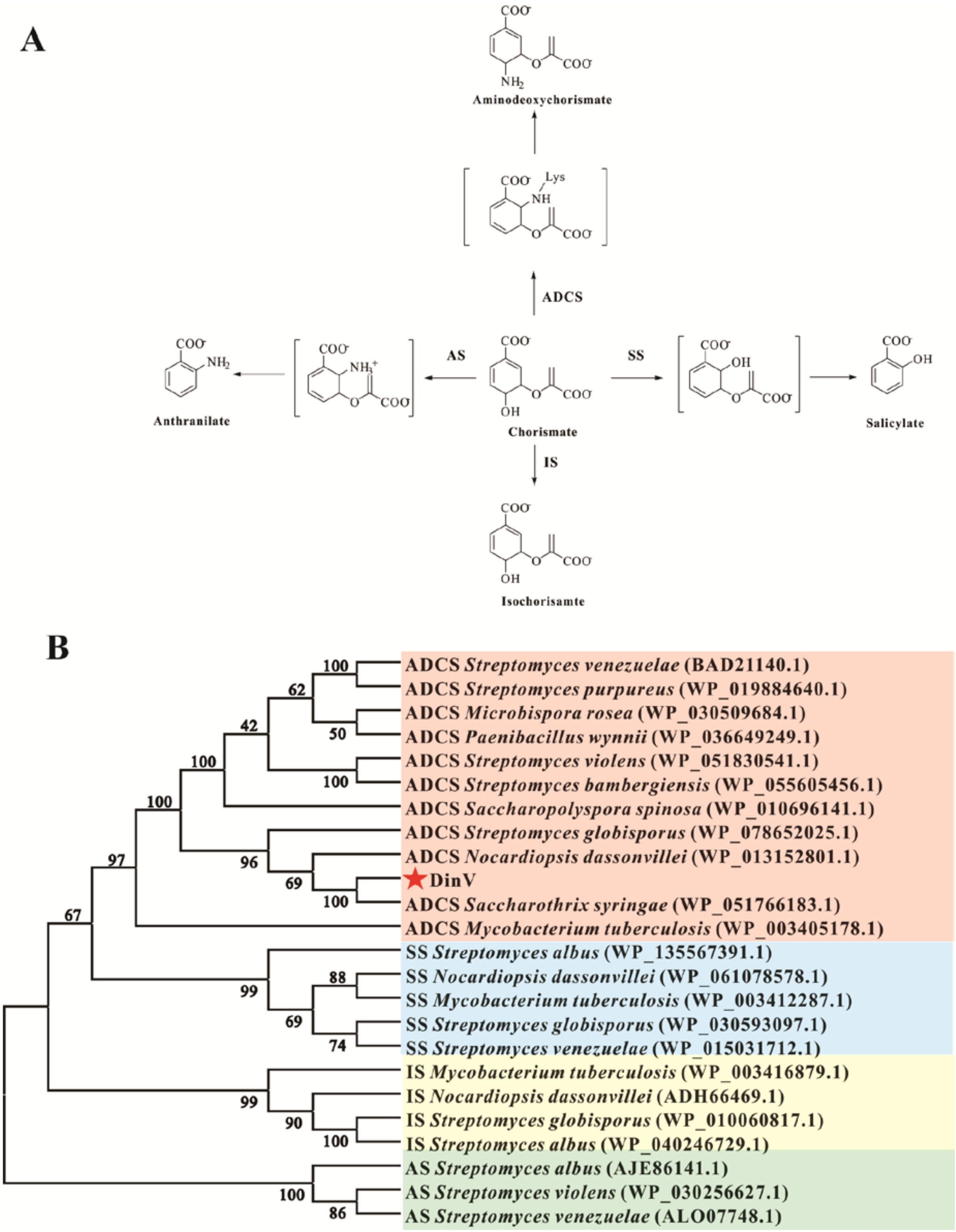

Figure S3. Biochemical and phylogenetic analysis of DinV. (A) Proposed function of the four homologous chorismate enzymes ADCS, IS, SS, and AS. (B) Phylogenetic tree of DinV constructed by MEGA7 using neighbor-joining method based on amino acid sequence. Numbers shown on the branch are the bootstrap values.

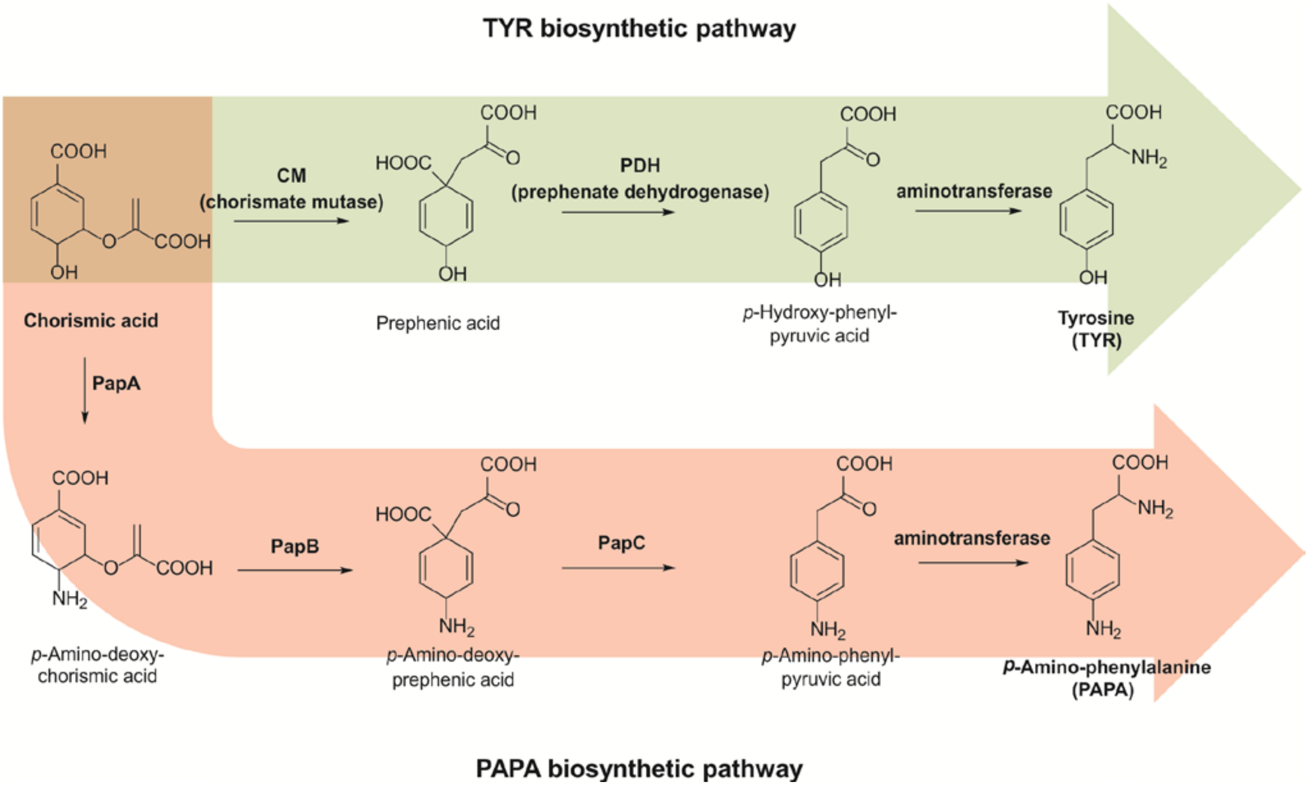

Figure S4. *p*-Amino-phenylalanine (PAPA) biosynthetic pathway is parallel to that of tyrosine (TYR).

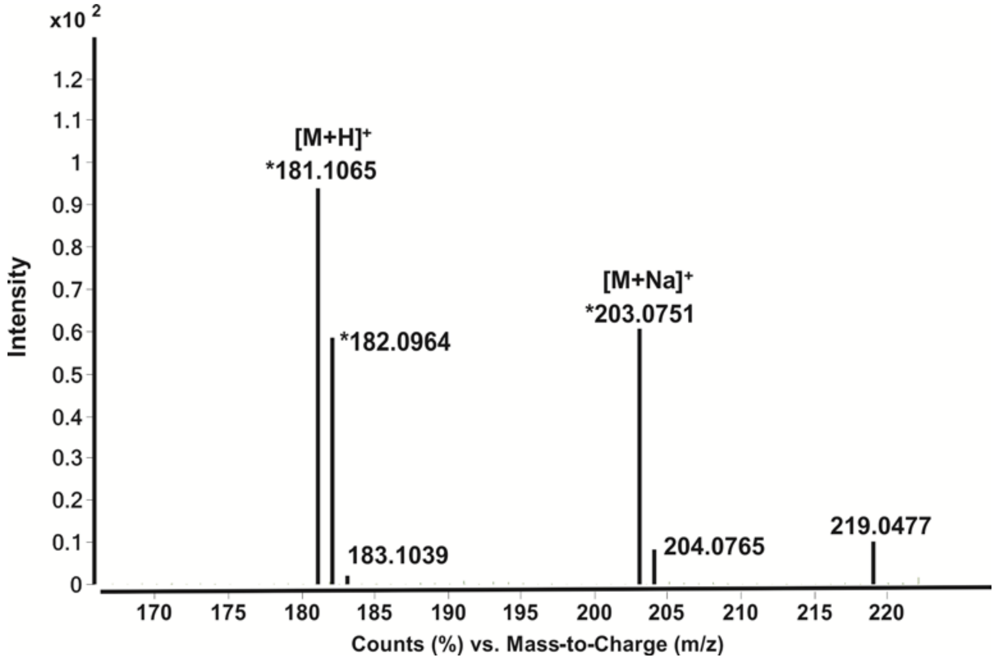

Figure S5. HR-ESI-MS analysis of PAPA.

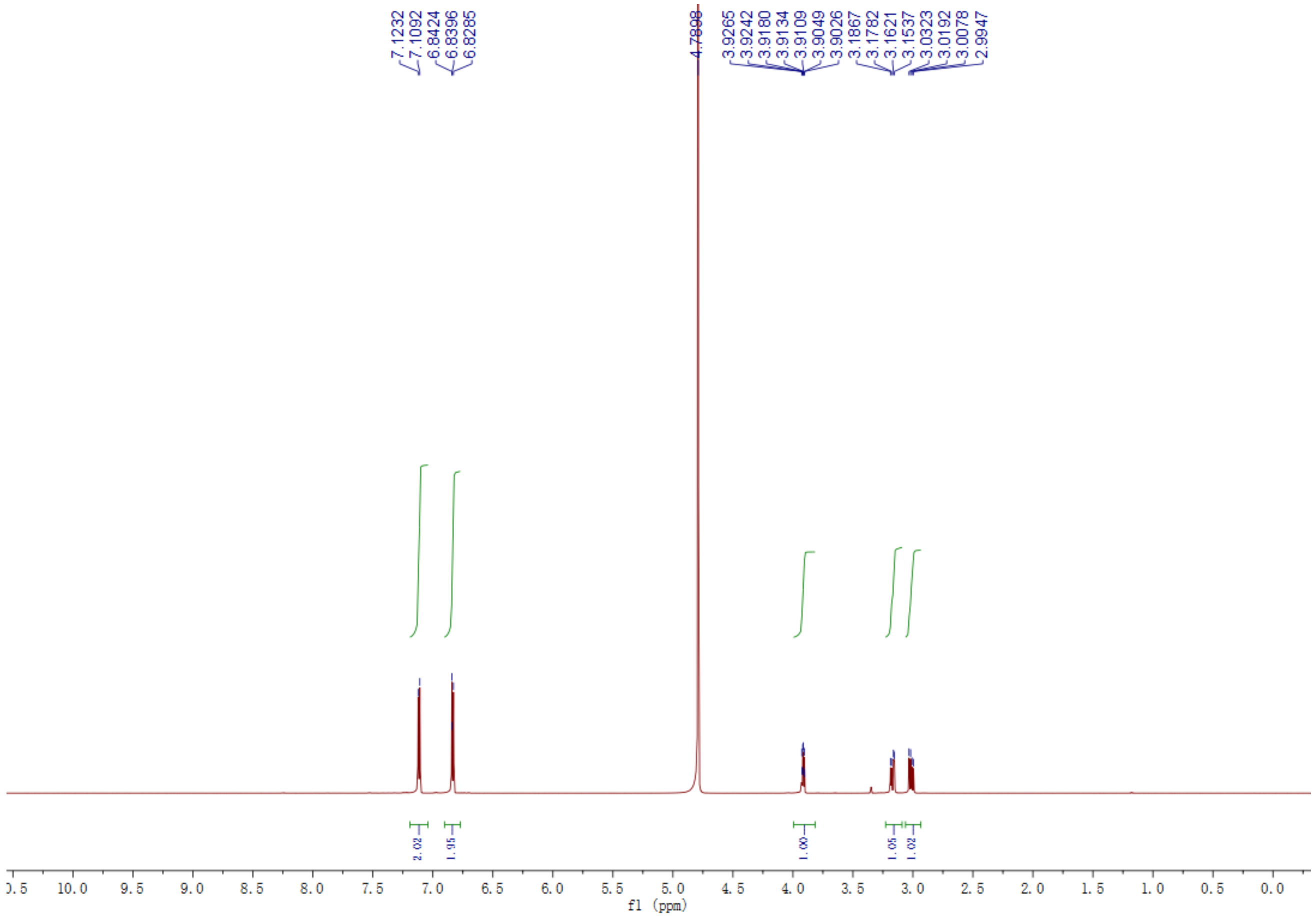

Figure S6. ^{1}H-NMR spectrum of PAPA in D_2O (600 MHz).

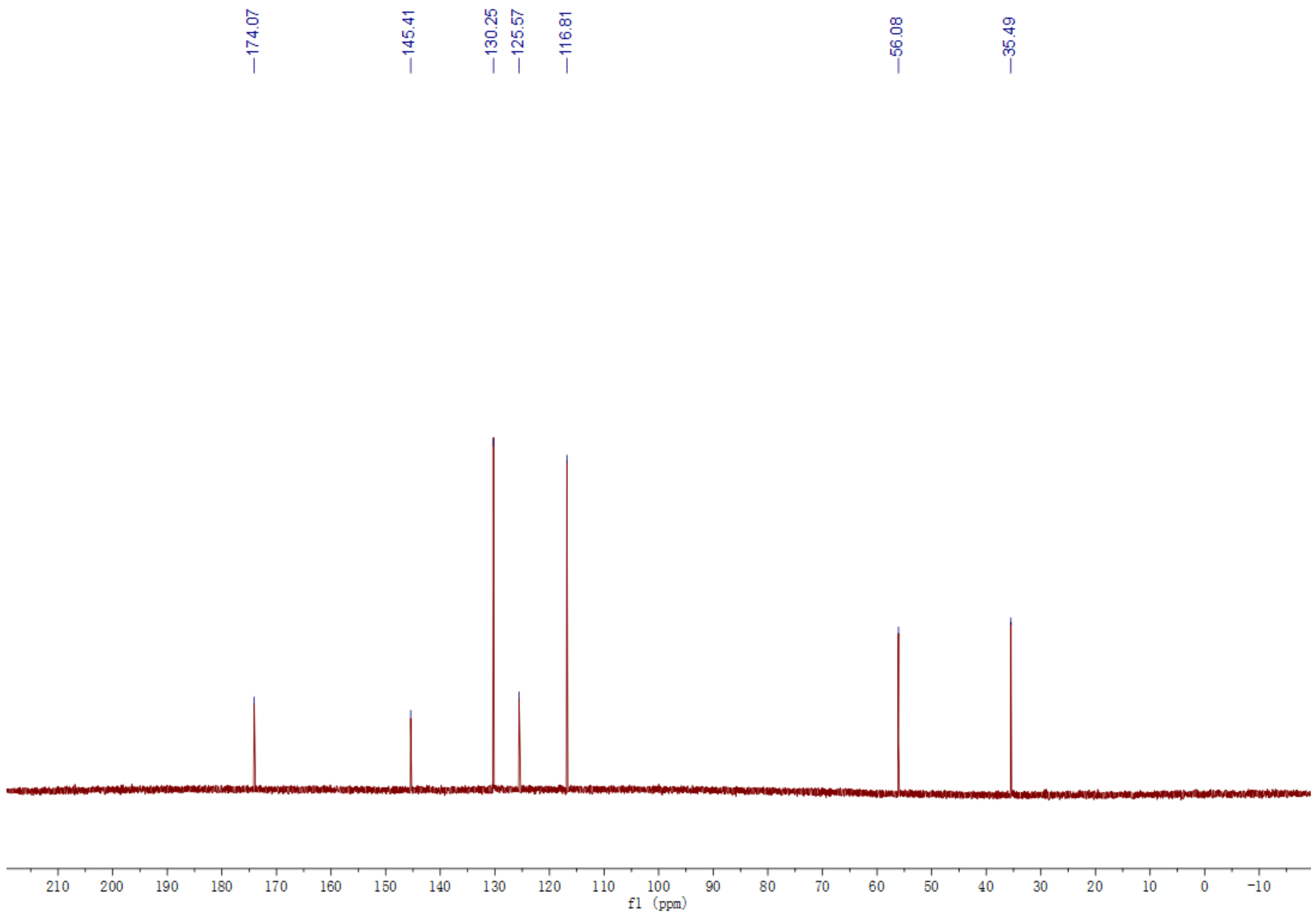

Figure S7. ^{13}C-NMR spectrum of PAPA in D_2O (150 MHz).

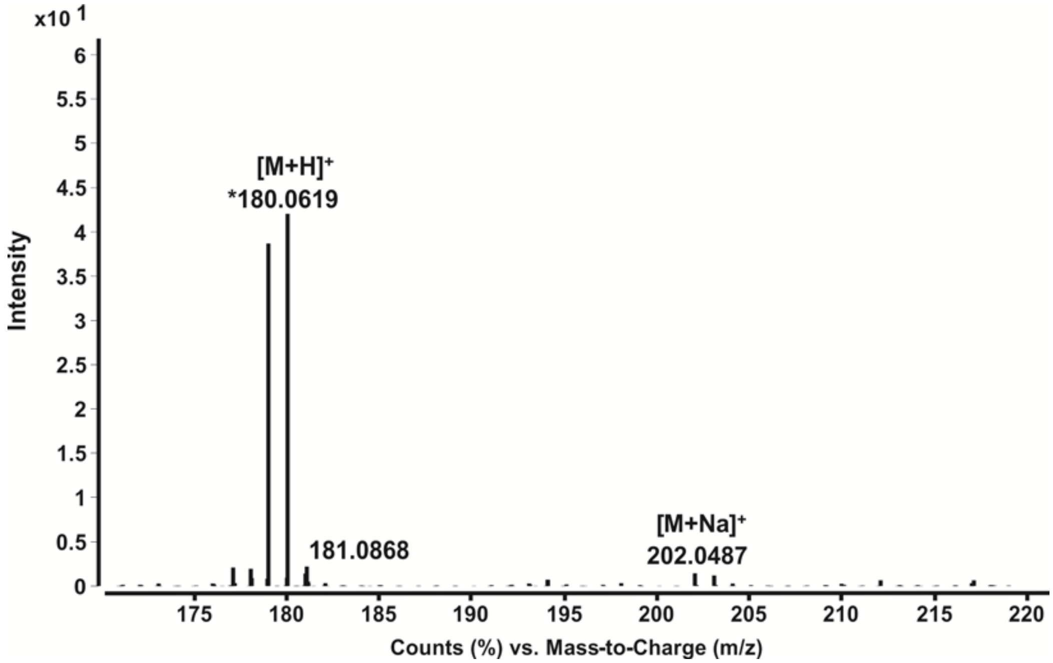

Figure S8. HR-ESI-MS analysis of PAPP.

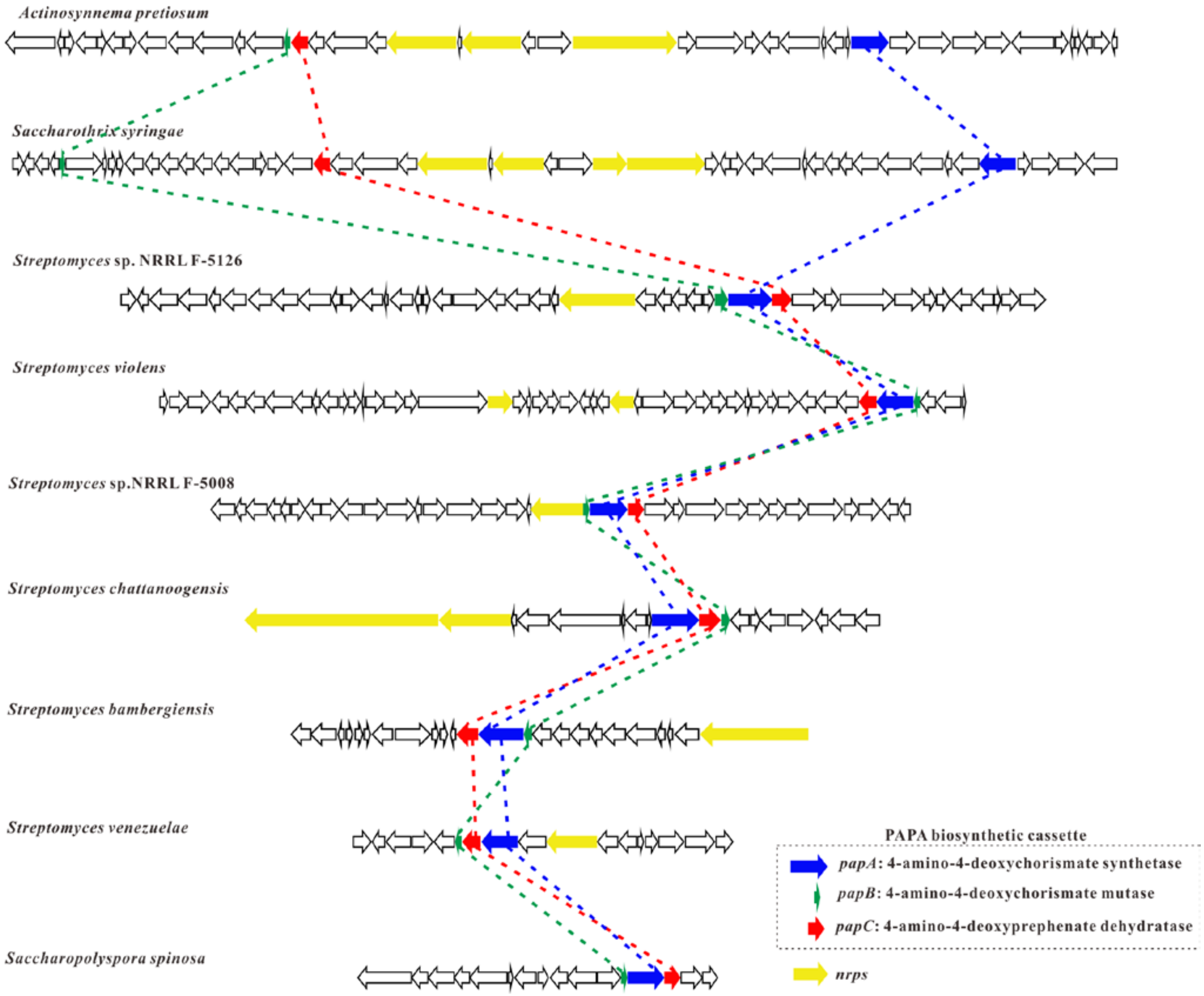

Figure S9. Discovery of bioactive natural products by mining PAPA biosynthetic gene cassette. The three-gene PAPA biosynthetic cassette was used to mine natural products containing PAPA building block.

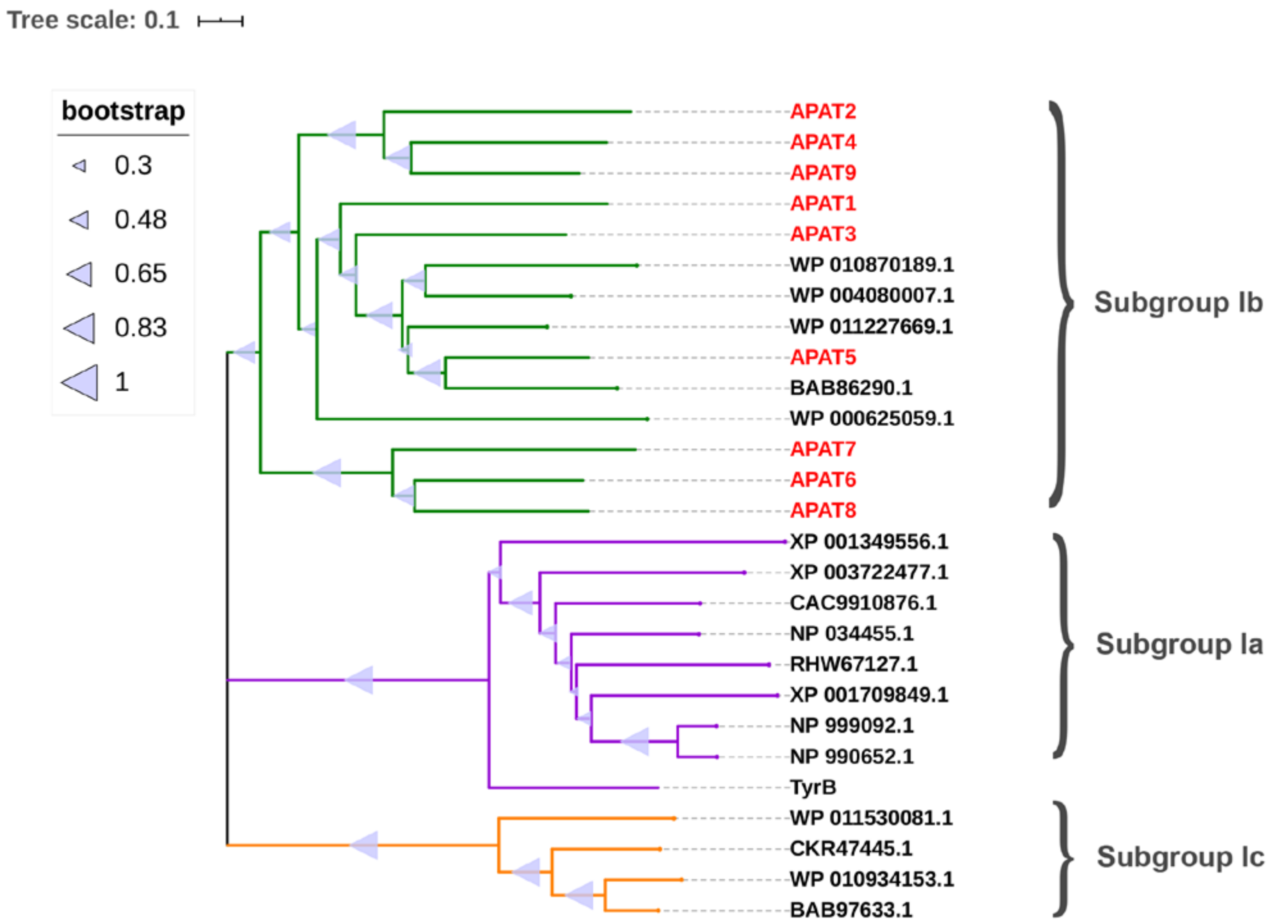

Figure S10. Phylogenetic analysis of APATs in strain DSM 44131^{T}. AT enzymes from different species of subgroup Ia, Ib, and Ic were chosen for the alignment. Phylogenetic tree was constructed by MEGA7 using neighbor-joining method based on amino acid sequence. WP_011530081.1: *Deinococcus geothermalis*; CKR47445.1: *Mycobacterium tuberculosis*; WP_010934153.1: *Corynebacterium diphtheria*; WP_000625059.1: *Staphylococcus aureus*; WP_010870189.1: *Methanococcus jannaschii*; BAB86290.1: *Phormidium lapideum*; WP_004080007.1: *Thermotoga maritima*; WP_011227669.1: *Thermus thermophiles*; XP_001349556.1: *Plasmodium falciparum*; XP_003722477.1: *Leishmania major*; CAC9910876.1: *Saccharomyces cerevisiae*; XP_001709849.1: *Giardia lamblia*; BAB97633.1: *Corynebacterium glutamicum*; NP_999092.1: *Sus scrofa*; RHW67127.1: *Trypanosoma brucei brucei*; NP_034455.1: *Mus musculus*; NP_990652.1: *Gallus gallus*.

Reference

He, Y., Wang, Z., Bai, L., Liang, J., Zhou, X., and Deng, Z. (2010). Two pHZ1358-derivative vectors for efficient gene knockout in streptomyces. *Journal of microbiology and biotechnology* 20(4), 678-682. doi: 10.4014/jmb.0910.10031.

Kieser, H.M., Kieser, T., and Hopwood, D.A. (1992). A combined genetic and physical map of the *Streptomyces coelicolor* A3(2) chromosome. *Journal of bacteriology* 174(17), 5496-5507. doi: 10.1128/jb.174.17.5496-5507.1992.

Tanida, S., Hasegawa, T., Muroi, M., and Higashide, E. (1980). Dnacins, new antibiotics. I. Producing organism, fermentation, and antimicrobial activities. *J Antibiot (Tokyo)* 33(12), 1443-1448. doi: 10.7164/antibiotics.33.1443.
